# Prognostic Value of Biomarkers in COVID-19: Associations with Disease Severity, Viral Variants, and Comorbidities—A Retrospective Observational Single-Center Cohort Study

**DOI:** 10.3390/life15040634

**Published:** 2025-04-10

**Authors:** Zoran Barušić, Kristian Bodulić, Sanja Zember, Renata Laškaj, Rok Čivljak, Ivan Puljiz, Ivan-Christian Kurolt, Željka Mačak Šafranko, Lidija Cvetko Krajinović, Petra Svoboda Karić, Alemka Markotić

**Affiliations:** 1 University Hospital for Infectious Diseases “Dr. Fran Mihaljević”, 10 000 Zagreb, Croatia; zbarusic@bfm.hr (Z.B.); kbodulic@bfm.hr (K.B.); szember@bfm.hr (S.Z.); rlaskaj@bfm.hr (R.L.); rcivljak@bfm.hr (R.Č.); ipuljiz@bfm.hr (I.P.); ikurolt@bfm.hr (I.-C.K.); zmacak@bfm.hr (Ž.M.Š.); lcvetko@bfm.hr (L.C.K.); psvoboda@bfm.hr (P.S.K.); 2 School of Medicine, University of Zagreb, 10 000 Zagreb, Croatia; 3 Faculty of Medicine, Catholic University of Croatia, 10 000 Zagreb, Croatia; 4 Faculty of Medicine, University of Rijeka, 51 000 Rijeka, Croatia

**Keywords:** COVID-19, biomarkers, SARS-CoV-2 variants, vaccination, comorbidities

## Abstract

Coronavirus disease (COVID-19) exhibits a wide spectrum of clinical severity and has been associated with specific biomarkers linked to disease progression and outcomes. This retrospective study analyzed sera from 1222 adult COVID-19 patients hospitalized at the University Hospital for Infectious Diseases in Croatia. We examined the association between several laboratory biomarker levels measured at patient admission and disease severity, fatal outcomes, viral variants and clinical parameters. Deceased patients and surviving patients with severe COVID-19 exhibited significantly elevated levels of several biomarkers on admission, including hs-troponin T, N-terminal pro-brain natriuretic peptide, creatine kinase, C-reactive protein, procalcitonin, interleukin-6, lactate dehydrogenase, lactate, urea and creatinine. Random forest models identified lymphocyte percentage, D-dimers, and hs-troponin T as the most important biomarkers for fatal outcome prediction, achieving 84.1% accuracy. Patients infected with the Delta SARS-CoV-2 variant exhibited significantly higher levels of proinflammatory, cardiac and renal biomarkers. Vaccination correlated with reduced proinflammatory parameters and higher lymphocyte proportions. Hypertension, chronic renal disease and diabetes were associated with increased cardiac, renal and metabolic biomarker levels, respectively. These findings highlight the association of several laboratory biomarkers with COVID-19 severity, viral variants, vaccination status and comorbidities, potentially offering prognostic insights into COVID-19 outcomes.

## 1. Introduction

Coronaviruses are a diverse family of viruses known to cause respiratory illnesses in humans, ranging from mild to severe disease. In late 2019, a novel coronavirus, later named severe acute respiratory syndrome coronavirus 2 (SARS-CoV-2), was identified in Wuhan, China. This led to the outbreak of coronavirus disease (COVID-19), which rapidly evolved into a pandemic with considerable mortality [1]. SARS-CoV-2 has been detected in various animal species, especially bats, and multiple instances of transmission from animals to humans have been documented. As a result, COVID-19 is recognized as a zoonotic disease [2]. Since the emergence of SARS-CoV-2, the virus has undergone numerous mutations, giving rise to several genetically distinct variants. These variants often show changes in transmissibility, immune evasion and disease severity. Notably, variants of concern such as B.1.1.7 (Alpha), B.1.617.2 (Delta) and B.1.1.529 (Omicron) have been linked to varying COVID-19 clinical manifestations, with the B.1.617.2 variant exhibiting higher disease severity and worse clinical outcomes [3,4].

COVID-19 exhibits a wide spectrum of clinical severity, ranging from mild to life-threatening conditions. A significant proportion of infected individuals are asymptomatic, showing no pronounced signs of illness. While the disease primarily affects the respiratory system, evidence indicates that COVID-19 is a multisystemic condition involving complex pathophysiological mechanisms. Severe COVID-19 cases often result from multifactorial processes, including systemic inflammation triggered by an uncontrolled immune response (cytokine storm), coagulopathy and other complications. Recovery from infection with a particular viral variant does not guarantee immunity, as both reactivations and reinfections have been extensively documented [5,6].

Multiple studies on COVID-19 have sought to identify key laboratory parameters that predict disease severity. A meta-analysis by Moutchi et al. revealed distinct dynamics of markers associated with innate and adaptive immune responses, as well as markers indicating damage to specific organ systems in patients with severe forms of COVID-19. Notably, interleukin-6, lymphocyte count, hs-troponin T, ferritin and D-dimers emerged as particularly significant indicators of COVID-19 severity [7]. A similar study found a strong association between COVID-19 severity and elevated levels of C-reactive protein (CRP), procalcitonin, interleukin-6, neutrophils, hs-troponin T, lactate dehydrogenase (LDH), ferritin and D-dimers, along with decreased levels of albumin, lymphocytes and platelets [8]. Furthermore, an increased neutrophil-to-lymphocyte ratio and a decreased lymphocyte-to-monocyte ratio at disease onset were found to be highly predictive of disease progression and mortality. These ratios also positively correlated with the modified early warning score, further highlighting their prognostic value [9]. Overall, most studies consistently demonstrated that specific biomarker dynamics are valuable for predicting the course and outcomes of COVID-19, providing critical insights into disease progression [7,8,9,10].

In this retrospective single-center cohort study, we analyzed data from 1222 adult patients diagnosed with COVID-19 hospitalized at the University Hospital for Infectious Diseases “Dr. Fran Mihaljević”, a tertiary care hospital and the Referral Center for Infectious Diseases in Croatia. This analysis focused on examining the relationship between patient outcomes and laboratory data. Biomarker levels measured on patient admission were utilized to build a machine learning model that accurately classified patients by clinical outcome. Additionally, we investigated the association of laboratory findings with viral variants and several clinical parameters.

## 2. Materials and Methods

This study analyzed 1222 adult COVID-19 patients hospitalized at the University Hospital for Infectious Diseases “Dr. Fran Mihalević”, which is the leading institution for the treatment of COVID-19 in Croatia. The analyzed patients were hospitalized between October 2020 and December 2021. Patients were recruited randomly using a random number generator, with a 3:1 ratio favoring surviving patients. This ensured a higher representation of deceased patients, allowing a detailed investigation of the associations between COVID-19 biomarkers and poor outcomes. Viral variants were inferred from the patient admission date, as different time periods coincided with specific SARS-CoV-2 variants circulating in the Croatian population (2 October 2020–31 January 2021: B.1 variant; 12 April 2021–15 June 2021: B.1.1.7 variant; 15 August 2021–31 December 2021: B.1.617.2. variant) [11]. All of the recruited patients had a confirmed SARS-CoV-2 infection via real-time polymerase chain reaction (RT-PCR) of nasopharyngeal swab samples. Blood samples for biochemical analysis were collected on patient admission and routinely during hospitalization. Analyzed biochemical and hematological parameters (biomarkers) included serum levels of sodium, potassium, chloride, hs-troponin T, N-terminal pro-brain natriuretic peptide (NT-proBNP), creatine kinase (CK), creatine kinase myocardial band (CKMB), ferritin, CRP, procalcitonin, interleukin-6, gamma-glutamyl transferase (GGT), alkaline phosphatase (ALP), bilirubin, LDH, glucose, albumin, total proteins, urea, creatinine, hemoglobin, fibrinogen, D-dimers and complete blood count. Demographic and clinical parameters, as well as vaccination history, were collected from patients’ medical records following standard ethical guidelines. A patient was considered fully vaccinated upon receiving at least either one injection of the Ad26.CoV2-S vaccine (Janssen) or two injections of the ChAdOx1-S vaccine (AstraZeneca) and/or RNA vaccines (tozinameran [Pfizer/BioNTech] and elasomeran [Moderna]), with the last injection administered at least 14 days before the positive PCR test result. Disease severity was determined during the most severe disease period according to the national treatment guidelines, published by the Ministry of Health of the Republic of Croatia [12]. The criteria for disease severity classification employed on the analyzed patients are shown in Table S1. This research was approved by the Ethics Committee of the University Hospital for Infectious Diseases (protocol code 01–673–5–2021, date of approval 10 May 2021).

Numerical variables were represented using medians, interquartile ranges (IQR) and ranges, while categorical variables were expressed with counts and percentages. Numerical variables between two patient groups were compared using the two-sample *t*-test in the case of normally distributed variables and the Mann–Whitney U test in the case of nonparametric distributions. Normally distributed numerical variables were compared in more than two patient groups with the analysis of variance (ANOVA), while nonparametric distributions were compared with the Kruskal–Wallis test, followed by Tukey-Kramer and Dunn post-hoc tests, respectively. Using the analyzed biomarkers, Random Forest models were utilized to classify patients according to disease outcome. Hyperparameter tuning was guided using the ten-fold cross-validation. As a result, the number of trees was set to 10,000 and four variables were considered at every split point. Variables included in the random forest analysis were chosen with the best subset selection method on the whole dataset, minimizing the ten-fold cross-validation error rate. The performance of the Random Forest models was evaluated on the test set following an 80:20 train–test dataset split. Confidence intervals (CI) for the area under the curve (AUC) were calculated using DeLong’s method. The predictor importance was estimated by calculating the average Gini index reduction for every predictor. Missing values of laboratory parameters (an average of 4.1% of missing values per parameter) were imputed using the MissForest R package. The association of the analyzed biomarkers with demographic and clinical parameters of interest was assessed using multiple linear regression. Residual distribution normality was evaluated by residual versus fit plots and quantile–quantile plots. All statistical tests were two-tailed with a significance level of 0.05. The *p*-values were corrected for multiple testing with the Benjamini-Hochberg method. Statistical analysis and data visualization were performed in R (version 4.4.2.) [13].

## 3. Results

### 3.1. Baseline Demographic and Clinical Characteristics

We analyzed a cohort of 1222 adult COVID-19 patients hospitalized at the University Hospital for Infectious Diseases between October 2020 and December 2021 (Table 1). A total of 797 (65.2%) patients were male, with a median age of 66 years (IQR 55–76 years, range: 23–98 years). A minority (178, 14.6%) of patients had been vaccinated before hospitalization and 1040 (85.1%) patients suffered from comorbidities. The most prevalent comorbidities included hypertension (738, 60.4%), cardiovascular diseases (320, 26.2%), diabetes (266, 21.8%) and chronic renal disease (86, 7.0%). A total of 71 (6.1%) patients were immunodeficient, with the most common causes including immunosuppressive cancer treatment (N = 41), primary immunodeficiency (N = 14) and immunodeficiency accompanied by solid organ transplant (N = 4). The median duration of disease on admission was 9 days (IQR 7–12, range 0–26 days), and the median hospitalization length was 10 days (IQR 7–16 days). A total of 297 (24.3%) patients were hospitalized in the intensive-care unit (ICU), while 229 (18.7%) patients underwent mechanical ventilation. When considering disease severity, we stratified the analyzed COVID-19 patients into five categories: surviving patients with mild (N = 123, 10.1%), moderate (N = 498, 40.8%), severe (N = 210, 17.2%) and critical COVID-19 (N = 70, 5.7%), as well as deceased patients (N = 314, 25.7%).

### 3.2. Association of the Analyzed Biomarkers with COVID-19 Severity and Fatal Outcomes

**Figure 1:**
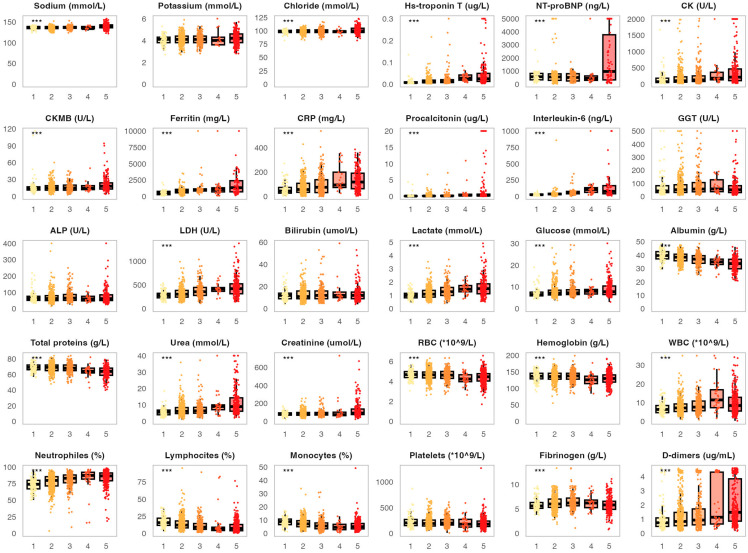
Distribution of the analyzed biomarkers measured on patient admission in patients with different COVID-19 severity. The boxes show the median and interquartile range of the distribution, while the whiskers extend to the minimum and maximum nonoutlier values of the distribution. Points denote individual participants. *** *p* < 0.001 (ANOVA or Kruskal–Wallis test depending on the biomarker distribution normality, *p*-values adjusted for multiple comparisons with the Benjamini-Hochberg method). 1 = surviving patients with mild COVID-19, 2 = surviving patients with moderate COVID-19, 3 = surviving patients with severe COVID-19, 4 = surviving patients with critical COVID-19, 5 = deceased patients. NT-proBNP = N-terminal pro-brain natriuretic peptide, CK = creatine kinase, CKMB = creatine kinase myocardial band, CRP = C-reactive protein, GGT = gamma-glutamyl transferase, ALP = alkaline phosphatase, LDH = lactate dehydrogenase, RBC = red blood cell, WBC = white blood cell.

The key part of this study was analyzing the association between laboratory parameters on admission and COVID-19 severity. In this context, we analyzed the distribution of the chosen biomarkers measured on admission in patient groups stratified by disease severity (Figure 1). Several biomarkers displayed significantly higher values among surviving patients with increasing COVID-19 severity (mild, moderate, severe and critical). This included cardiac parameters such as hs-troponin T (medians 0.007, 0.011, 0.015 and 0.025 µg/L, *p* < 0.001) and CK (medians 91, 102, 140 and 220 U/L). This trend was also found in proinflammatory biomarkers like ferritin (medians 522, 786, 977 and 1046 mg/L, *p* < 0.001), CRP (medians 72.5, 81.2, 95.6 and 112.1 mg/L, *p* < 0.001), procalcitonin (medians 0.11, 0.18, 0.21 and 0.52 µg/L, *p* < 0.001) and interleukin-6 (medians 27.6, 49.8, 80.6 and 106.3 ng/L, *p* < 0.001). A significant association with COVID-19 severity was also found for LDH (medians 290, 315, 380 and 472 U/L, *p* < 0.001), lactate (medians 1.1, 1.3, 1.5 and 1.7 mmol/L), urea (medians 5.0, 7.1, 7.5 and 10.4 mmol/L) and D-dimers (medians 0.81, 0.92, 1.01 and 1.30 µg/mL, *p* < 0.001). On the other hand, surviving patients with higher COVID-19 severity showed lower monocyte (medians 9.8%, 8.6%, 8.5% and 6.1%, *p* = 0.019) and lymphocyte proportions (medians 20.1%, 12.8%, 10.1% and 7.9%, *p* < 0.001), as well as slightly lower levels of albumin (medians 41, 39, 37 and 34 g/L, *p* < 0.001). Generally, deceased patients had similar biomarker levels on admission when compared to surviving patients with critical COVID-19 (Figure 1). However, two parameters were significantly higher in deceased patients, including NT-proBNP (medians 991.5 and 444.5 ng/L, *p* < 0.001) and ferritin (medians 1328 and 1046 mg/L, *p* = 0.001).

All of the observed associations between biomarker levels recorded at patient admission and COVID-19 severity were also found when analyzing biomarker levels during the most severe disease period (Figure S1). In addition, several other parameters measured during the most severe disease period were significantly increased in deceased patients and surviving patients with critical disease severity when compared to patients with milder disease forms. This included ALP (medians 101.1 U/L, 99.8 U/L and 80.1 U/L, *p* < 0.001), bilirubin (medians 35, 34 and 21 µmol/L, *p* < 0.001) and creatinine (medians 200.1, 140.9 and 130.5 µmol/L, *p* < 0.001).

### 3.3. Predictive Ability of the Analyzed Biomarkers for COVID-19 Outcomes

**Figure 2:**
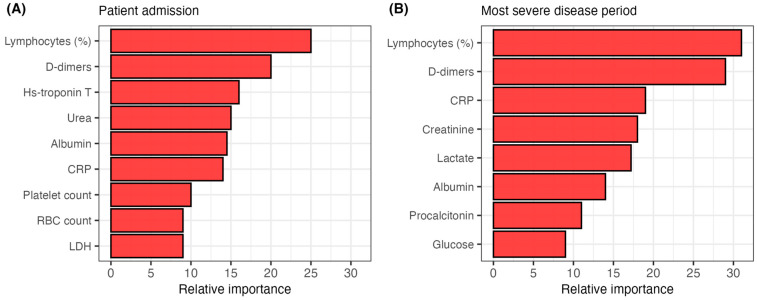
The relative importance of predictors utilized in the Random Forest models for classifying patients by disease outcome. (**A**) model classifying patients by outcome using biomarkers measured on admission. (**B**) model classifying patients by outcome using biomarkers measured during the most severe disease period. CRP = C-reactive protein, RBC = red blood cell, LDH = lactate dehydrogenase.

We also assessed the potential of the analyzed biomarkers for predicting fatal outcomes of COVID-19 patients. We tested two biomarker sets: biomarkers recorded on patient admission and during the most severe disease period. Biomarker predictive abilities were tested with Random Forest models, allowing for an estimate of biomarker importance in predicting fatal outcomes (Figure 2). The model using biomarkers measured at patient admission exhibited an accuracy of 84.1% (sensitivity 91.2%, specificity 71.6%, AUC = 0.89, 95% CI 0.86–0.93) (Figure S2). Biomarkers included in this model were: lymphocyte proportion, D-dimers, hs-troponin T, urea, albumin, CRP, platelet count, RBC count and LDH (Figure 2A). The model utilizing biomarkers measured during the most severe disease period showed an accuracy of 91.8% (sensitivity 93.9%, specificity 86.9%, AUC = 0.93, 95% CI 0.90–0.95) (Figure S2). Biomarkers determined during the most severe disease period used in this model were: lymphocyte proportion, D-dimers, CRP, creatinine, lactate, albumin, procalcitonin and glucose (Figure 2B).

### 3.4. Association of the Analyzed Biomarkers with Viral Variants

**Figure 3:**
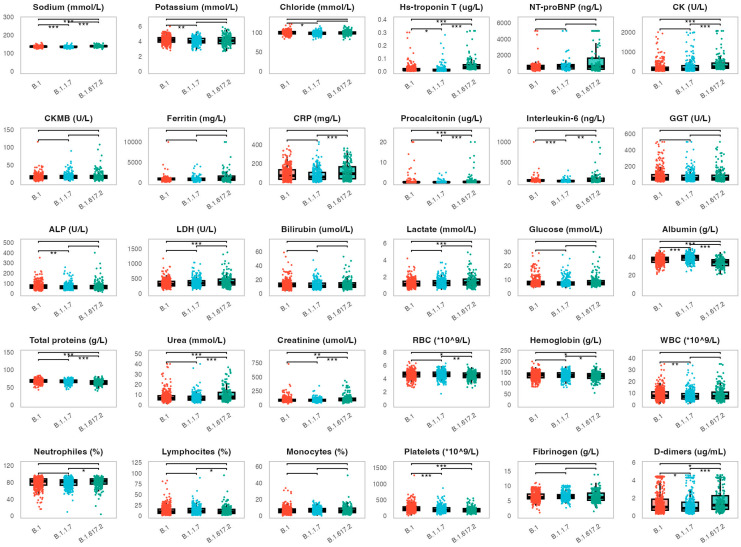
Distribution of the analyzed biomarkers measured on patient admission in patients infected with the B.1., B.1.1.7 and B.1.617.2 viral variants. The boxes show the median and interquartile range of the distribution, while the whiskers extend to the minimum and maximum nonoutlier values of the distribution. Points denote individual participants. *** *p* < 0.001, ** *p* < 0.01, * *p* < 0.05 (ANOVA with Tukey-Kramer post-hoc test or Kruskall–Wallis with Dunn’s post-hoc test depending on the biomarker distribution normality, *p*-values adjusted for multiple comparisons with the Benjamini-Hochberg method). NT-proBNP = N-terminal pro-brain natriuretic peptide, CK = creatine kinase, CKMB = creatine kinase myocardial band, CRP = C-reactive protein, GGT = gamma-glutamyl transferase, ALP = alkaline phosphatase, LDH = lactate dehydrogenase, RBC = red blood cell, WBC = white blood cell.

Next, we assessed the potential correlation between the analyzed biomarkers and viral variants (Figure 3). We evaluated biomarker levels measured on admission in 397 patients infected with the B.1 variant, 293 patients with the B.1.1.7 variant and 330 patients with the B.1.617.2 variant. Patients infected with the B.1.617.2 variant showed significantly higher levels of several biomarkers when compared to patients infected with B.1 and B.1.1.7 variants. This primarily included cardiac parameters (hs-troponin T: medians 0.035, 0.011 and 0.009 µg/L, *p* < 0.001, CK: medians 201, 135 and 122 U/L, *p* < 0.001). Similar trends were found for procalcitonin (medians 0.25, 0.14 and 0.13 g/L, *p* < 0.001), as well as for urea (medians 7.0, 6.4 and 6.2 mmol/L, *p* < 0.001) and D-dimers (medians 1.20, 0.98 and 0.81 µg/mL, *p* < 0.001). Furthermore, patients infected with the B1.617.2 variant exhibited significantly lower levels of albumin when compared to other variants (medians 34, 39 and 40 g/L, *p* < 0.001). CRP levels were significantly higher in patients infected with the B1.617.2 variant compared to the B.1.1.7 variant (medians 91.8 and 59.6 mg/L, *p* < 0.001). Similarly, interleukin-6 levels were similar in patients infected with B.1.617.2 and B.1 variants (medians 50 and 47 ng/L), but significantly higher when compared to patients infected with the B.1.1.7 variant (median 34 ng/L, *p* = 0.004, *p* < 0.001, respectively).

The observed associations between biomarker levels determined on patient admission and viral variants were further evaluated with multiple linear regression (Table 2). We fit separate regression models for each biomarker using the following demographic and clinical parameters: age, sex, viral variant, vaccination status and comorbidities. This allowed us to examine the effect of viral variants on each biomarker while controlling for the effects of the stated covariates. The analysis confirmed the significant associations described in Figure 3.

### 3.5. Association of the Analyzed Biomarkers with Vaccination Status and Comorbidities

The multiple linear regression models presented in 3.4 were also utilized to assess the impact of vaccination and comorbidities on the biomarkers measured at patient admission. We found a significant association between vaccination and proinflammatory parameters, with vaccinated patients on average having 1.79 times (95% CI 1.20–2.39, *p* < 0.001) lower levels of procalcitonin and 1.54 times (95% CI 1.05–2.02, *p* = 0.048) higher lymphocyte proportions.

The association between the analyzed biomarkers and the most prevalent comorbidities is shown in Table 3. We found a significant correlation between cardiovascular comorbidities and cardiac markers during acute COVID-19. Patients with hypertension exhibited 1.38 times (95% CI 1.31–1.45, *p* < 0.001) higher levels of hs-troponin T, 1.40 times (95% CI 1.23–1.57, *p* < 0.001) higher levels of NT-proBNP and 1.32 times (95% CI 1.24–1.40, *p* < 0.001) higher levels of CK. Similar correlations were found for patients with cardiovascular diseases, along with 1.33 times (95% CI 1.10–1.56, *p* = 0.038) higher levels of fibrinogen and 1.40 times (95% CI 1.15–1.64, *p* = 0.027) higher levels of D-dimers. We also recorded higher levels of a wide range of metabolic markers in COVID-19 patients with diabetes. This included 1.60 times (95% CI 1.34–1.86, *p* < 0.001) higher levels of ALP, 1.55 times (95% CI 1.21–1.79, *p* = 0.017) higher levels of LDH and 1.50 times (95% CI 1.25–1.74, *p* = 0.001) higher levels of glucose. Patients with chronic renal disease displayed higher cardiac and renal biomarker levels during acute COVID-19, including 1.50 times (95% CI 1.06–1.96, *p* = 0.049) higher levels of hs-troponin T, 1.64 times (95% CI 1.20–2.08, *p* = 0.021) higher levels of urea and 1.70 times (95% CI 1.35–2.05, *p* < 0.001) higher levels of creatinine. Immunodeficient patients showed higher levels of proinflammatory biomarkers such as CRP (fold change 1.48, 95% CI 1.08–1.89, *p* = 0.040) and interleukin-6 (fold change 1.50, 95% CI 1.08–1.91, *p* = 0.043), as well as lower lymphocyte proportion (fold change 0.60, 95% CI 0.25–0.95, *p* = 0.046).

## 4. Discussion

The present study provides a comprehensive analysis of the association between laboratory biomarkers and COVID-19 severity, viral variants and several clinical parameters in a large cohort of patients hospitalized with COVID-19 in a single tertiary care hospital in Croatia. Our findings reinforce the prognostic utility of several biomarkers in predicting COVID-19 severity and outcomes, adding to the large amount of evidence that highlights the complexity of COVID-19 immunopathology [4,5,6,7,8,9,10]. The results of this study reveal a strong association of several biomarkers recorded on patient admission and during the most severe disease period with COVID-19 severity and outcome. This includes proinflammatory markers, such as CRP, procalcitonin and interleukin-6, highlighting the important role of systemic inflammation in COVID-19 progression. These findings are consistent with the cytokine storm phenomenon described in severe COVID-19 cases by several studies [7,8,14]. The cytokine storm represents an exaggerated immune response characterized by the overproduction of proinflammatory cytokines such as interleukin-6, tumor necrosis factor-alpha and interleukin-1 beta. This can lead to widespread tissue damage, endothelial dysfunction and organ failure, contributing to the high mortality observed in patients with severe COVID-19 [14,15]. Targeting the cytokine storm with immunomodulatory therapies has been shown to mitigate inflammation and improve outcomes in severe COVID-19 cases, highlighting the clinical relevance of proinflammatory biomarkers in monitoring COVID-19 progression and treatment [16,17]. Our findings also demonstrate the importance of lymphocyte proportion as a possible protective factor during SARS-CoV-2 infection. Lymphocyte depletion reflects immune system dysregulation and a systemic proinflammatory response during severe COVID-19, aligning with prior reports of lymphopenia as an adverse prognostic factor in COVID-19 patients, including longer hospitalization and poor outcomes [9,17]. Lymphopenia observed during severe COVID-19 may result from immune exhaustion caused by the cytokine storm or lymphocyte redistribution to the inflamed tissues [17]. All in all, the results further emphasize the role of immune system dysregulation in severe COVID-19.

The elevation of cardiac markers in deceased COVID-19 patients in our study could suggest a significant role of cardiac stress in the progression of severe COVID-19. This finding aligns with the results of several studies demonstrating increased levels of hs-troponin T, NT-proBNP and CK, while showing that SARS-CoV-2 infection can result in direct and indirect cardiovascular damage [18,19,20]. Mechanisms underlying these elevations may include viral-induced myocarditis, cytokine-mediated myocardial damage and microvascular thrombi resulting from the prothrombotic state often observed during COVID-19 progression [21,22]. Notably, significantly elevated D-dimer levels in patients with severe disease and deceased patients further support the occurrence of thrombotic events. Increased levels of proinflammatory, cardiac and thrombotic biomarkers in severe COVID-19 may be a consequence of the interplay between systemic inflammation, vascular dysfunction and cardiac injury, highlighting the complex pathophysiology of COVID-19 [21,22].

Our results also suggest an important role of liver and renal biomarkers in predicting COVID-19 severity and outcomes. The elevation of LDH could indicate a widespread hepatic tissue injury, while elevated lactate levels point towards tissue hypoxia and anaerobic metabolism, often seen in severe inflammation and organ dysfunction associated with COVID-19. These results align with previous research identifying hepatic injury as an important factor in COVID-19 progression [23]. Similarly, elevated renal markers like creatinine and urea indicate renal dysfunction, probably caused by systemic inflammation, immune-related kidney damage and potential virus-induced cytopathic effects [24]. This can contribute to acute kidney injury, which complicates the clinical course of severe COVID-19. Renal dysfunction in COVID-19 is often accompanied by cardiac events, with reduced cardiac output further impairing renal perfusion and exacerbating kidney injury. In the context of COVID-19, this may be aggravated by systemic inflammation, endothelial dysfunction and microvascular damage, all of which contribute to the high mortality observed in critically ill patients [24].

The Random Forest models presented in this study demonstrated significant predictive capability, with biomarkers measured on patient admission achieving an accuracy of 84.1% for classifying patient outcomes. Notably, the model was more accurate in classifying deceased patients (sensitivity 91.2%) than surviving patients (specificity 71.6%). The substantial predictive power of biomarkers measured on admission highlights the potential of early laboratory findings to guide risk stratification in COVID-19 patients. Moreover, the model utilizing biomarker levels measured during the most severe disease period displayed significantly higher accuracy (91.8%). Even though this model highlights the importance of a diverse set of biomarkers in COVID-19 outcome prediction, the potential application of this model in a clinical setting is less clear. While these results agree with several studies highlighting the potential usage of machine learning models in predicting COVID-19 severity [25,26], these models should be carefully evaluated on a larger and more diverse patient cohort.

Although this study encompassed a comprehensive set of routinely measured biomarkers, several analytes not analyzed in this research have been shown to correlate with COVID-19 severity. This includes a wide range of cytokines (such as interferon-γ, interferon-α, interleukin-8 and interleukin-10) and chemokines (such as chemokine ligands 9, 10 and 11), which act as important players in COVID-19 immunopathology [27,28]. This also includes soluble angiotensin-converting enzyme 2, the primary binding site of SARS-CoV-2, which was found to be significantly elevated in patients with fatal COVID-19 outcomes [29]. The inclusion of a wider range of biomarkers would potentially allow for even higher predictive power of machine learning models predicting COVID-19 severity.

This study also revealed significant associations between laboratory biomarkers measured on patient admission and viral variants. Patients infected with the B.1.617.2 (Delta) variant exhibited higher levels of proinflammatory, cardiac and renal biomarkers. This finding suggests a significant association between the Delta variant and COVID-19 severity, which is supported by previous research [3,4]. Vaccinated patients demonstrated lower levels of procalcitonin and higher lymphocyte percentages at admission, suggesting a protective effect against severe inflammation and immune dysfunction. This aligns with research showing that vaccination reduces the risk of severe COVID-19 and associated complications [30,31,32,33,34]. Additionally, our findings suggest an association of comorbidities on biomarker profiles on admission, with cardiovascular diseases, diabetes, and chronic renal disease significantly altering cardiac, renal, and metabolic parameters, respectively. Notably, these findings may not necessarily reflect the pathophysiological effects of COVID-19, but may be a direct consequence of the respective comorbidities. These associations go hand in hand with studies demonstrating worse COVID-19 prognosis in patients with multiple comorbidities [35,36]. Finally, our results revealed higher proinflammatory biomarker levels and lower lymphocyte levels in immunocompromised patients, which is in line with the report of higher COVID-19 severity in immunodeficient cohorts [37]. However, given the heterogeneity of the studied immunocompromised cohort, this finding should be validated by future research on COVID-19 severity in precisely defined immunodeficient groups.

We acknowledge several limitations of this study. While this study analyzed a relatively large cohort of patients, the focus on a single center in Croatia may limit generalizability to other populations and healthcare settings. However, it should be noted that the center served as the primary hospital for COVID-19 treatment for the whole country. Due to the retrospective design, this study identifies associations between biomarker levels and COVID-19 severity, without establishing direct causality. Furthermore, as the studied samples were collected between 2020 and 2021, several aspects of the analysis were hindered. This primarily included the analysis of SARS-CoV-2 variants and their association with laboratory biomarkers, as the given time frame did not allow for the evaluation of novel variants, such as Omicron. Moreover, the analyzed period also reflects on the vaccinated subgroup, which was relatively scarce and mainly comprised of patients vaccinated with two vaccine doses. Finally, the compared viral variants were not directly sequenced, but were inferred from the patient’s hospitalization date. However, this limitation should not introduce significant bias into the variant comparison results, as the analyzed viral variants represented the vast majority of sequenced SARS-CoV-2 isolates during the analyzed periods [11]. Despite these shortcomings, our study revealed significant associations between several biomarkers and COVID-19 severity, viral variants, vaccination status and comorbidities. Future research should validate these findings in prospective, multicenter studies that encompass a broader range of SARS-CoV-2 variants and vaccination statuses, exploring causal mechanisms and enhancing the generalizability and clinical applicability of these results.

## Figures and Tables

**Table 1:** Baseline demographic and clinical characteristics of patients hospitalized with COVID-19 (N = 1222).

Variable	N (%)/Median (IQR)
Sex (male/female)	797/425 (65.2%/34.8%)
Age (years)	66 (55–76)
Age groups (years)	
18–50	200 (16.4%)
51–65	396 (32.4%)
66–80	435 (35.6%)
>80	191 (15.6%)
Viral variant	
B.1 (Original)	397 (32.5%)
B.1.1.7 (Alpha)	293 (24.0%)
B.1.617.2 (Delta)	330 (27.0%)
Unknown *	202 (16.5%)
Fully vaccinated against COVID-19	178 (14.6%)
Number of comorbidities	2 (1–3)
Most common comorbidities	
Hypertension	738 (60.4%)
Cardiovascular diseases	320 (26.2%)
Diabetes	266 (21.8%)
Chronic renal disease	86 (7.0%)
Immunodeficiency	71 (6.3%)
Duration of disease on admission (days)	9 (7–12)
Duration of hospitalization (days)	10 (7–16)
ICU admission	297 (24.3%)
Mechanical ventilation	229 (18.7%)
Disease severity	
Surviving patients, mild COVID-19	123 (10.1%)
Surviving patients, moderate COVID-19	498 (40.8%)
Surviving patients. severe COVID-19	210 (17.2%)
Surviving patients, critical COVID-19	70 (5.7%)
Deceased patients	314 (25.7%)

* Patients infected during the transitional viral variant periods; ICU = intensive-care unit.

**Table 2:** The association between biomarkers measured on admission and viral variants. The given values represent the average fold change of the corresponding biomarker for each viral variant comparison. The fold changes were calculated using multiple linear regression models, adjusting for patient age, sex, vaccination status and comorbidities. Non–significant fold changes are not shown (/).

	Fold Change (95% CI), *p*-Value		
Biomarker	B.1.617.2 vs. B.1	B.1.617.2 vs. B.1.1.7	B.1. vs. B.1.1.7
Hs-troponin T	2.90 (2.67–3.13), <0.001	2.97 (2.71–3.24), <0.001	1.32 (1.09–1.55), 0.043
CK	1.67 (1.42–1.92), <0.001	1.75 (1.48–2.01), <0.001	/
Procalcitonin	1.51 (1.25–1.76), 0.004	1.50 (1.26–1.74), 0.003	/
Interleukin-6		1.60 (1.36–1.85), <0.001	1.58 (1.37–1.79), <0.001
Urea	1.38 (1.15–1.61), 0.018	1.42 (1.13–1.70), 0.020	/
D-dimers	1.35 (1.08–1.62), 0.034	1.48 (1.20–1.76), 0.006	1.28 (1.05–1.52), 0.048
Albumin	0.68 (0.48–0.88), 0.012	0.67 (0.46–0.89), 0.014	0.71 (0.52–0.91), 0.021

CK = creatine kinase.

**Table 3:** The association between biomarkers measured on admission and the most prevalent comorbidities The given values represent the average fold change of the corresponding biomarker for each comorbidity. The fold changes were calculated using multiple linear regression models, adjusting for patient age, sex, vaccination status and viral variants. Non–significant fold changes are not shown (/).

	Fold Change (95% CI), *p*-Value				
Biomarker	Hypertension N = 738	Cardiovascular Disease N = 320	Diabetes N = 266	Chronic Renal Disease N = 86	Immunodeficiency N = 71
Hs-troponin T	1.38 (1.31–1.45), <0.001	1.45 (1.22–1.68), 0.012	/	1.50 (1.06–1.96), 0.049	/
NT-proBNP	1.40 (1.23–1.57), <0.001	1.60 (1.30–1.90), 0.001	/	1.70 (1.15–2.25), 0.043	/
CK	1.32 (1.24–1.40), <0.001	1.38 (1.16–1.60), 0.034	/	/	/
CRP	/	/	/	/	1.48 (1.08–1.89), 0.040
Interleukin-6	/	/	/	/	1.50 (1.08–1.91), 0.043
ALP	/	/	1.60 (1.34–1.86), <0.001	/	/
LDH	/	/	1.55 (1.21–1.79), 0.017	/	/
Lactate	/	/	1.40 (1.10–1.70), 0.034	/	/
Glucose	/	/	1.50 (1.25–1.74), 0.001	/	/
Albumin	/	/	/	1.88 (1.50–2.26), <0.001	/
Total proteins	/	/	/	1.60 (1.20–2.01), 0.023	/
Urea	/	/	/	1.64 (1.20–2.08), 0.021	/
Creatinine	/	/	/	1.70 (1.35–2.05), <0.001	
Neutrophils (%)	/	/	/	/	1.45 (1.05–1.86), 0.048
Lymphocytes (%)	/	/	/	/	0.60 (0.25–0.95), 0.046
Fibrinogen	/	1.33 (1.10–1.56), 0.038	/	/	/
D-dimers	/	1.40 (1.15–1.64), 0.027	/	/	/

NT-proBNP = N-terminal pro-brain natriuretic peptide, CK = creatine kinase, CRP = C-reactive protein, ALP = alkaline phosphatase, LDH = lactate dehydrogenase.

## Data Availability

The data presented in this study are available on request from the corresponding author.

## Supplementary Materials

The following supporting information can be downloaded at: https://www.mdpi.com/article/10.3390/life15040634/s1, Table S1: Clinical criteria for COVID-19 severity classification according to Croatian treatment guidelines used in this study. Figure S1: Distribution of the analyzed biomarkers measured during the most severe disease period in patients with different COVID-19 severity. The boxes show the median and interquartile range of the distribution, while the whiskers extend to the minimum and maximum nonoutlier values of the distribution. Points denote individual participants. *** *p* < 0.001, ** *p* < 0.01, * *p* < 0.05 (ANOVA or Kruskal–Wallis test depending on the biomarker distribution normality, *p*-values adjusted for multiple comparisons with the Benjamini-Hochberg method). 1 = surviving patients with mild COVID-19, 2 = surviving patients with moderate COVID-19, 3 = surviving patients with severe COVID-19, 4 = surviving patients with critical COVID-19, 5 = deceased patients. NT-proBNP = N-terminal pro–brain natriuretic peptide, CK = creatine kinase, CKMB = creatine kinase myocardial band, CRP = C-reactive protein, GGT = gamma-glutamyl transferase, ALP = alkaline phosphatase, LDH = lactate dehydrogenase, RBC = red blood cell, WBC = white blood cell; Figure S2: Receiver operating characteristic (ROC) curves of random forest models utilizing subsets of laboratory biomarkers in classifying COVID-19 patients according to disease outcome. AUC = area under the curve.

## Abbreviations

The following abbreviations are used in this manuscript:

SARS-CoV-2	Severe acute respiratory syndrome coronavirus 2
COVID-19	Coronavirus Disease 2019
CRP	C-reactive protein
LDH	Lactate dehydrogenase
RT-PCR	Real-time polymerase chain reaction
NT-proBNP	N-terminal pro–brain natriuretic peptide
CK	Creatine kinase
CKMB	Creatine kinase myocardial band
GGT	Gamma-glutamyl transferase
ALP	Alkaline phosphatase
IQR	Interquartile range
ANOIVA	Analysis of variance
CI	Confidence interval
AUC	Area under the curve
ICU	Intensive-care unit
RBC	Red blood cell
WBC	White blood cell
