# Supplementary material for: Prognostic Value of Biomarkers in COVID-19: Associations with Disease Severity, Viral Variants, and Comorbidities—A Retrospective Observational Single-Center Cohort Study
Source: Life (Basel). 2025 Apr 10;15(4):634. doi: 10.3390/life15040634 (PMC12028838; doi:10.3390/life15040634)
Supplement: Supplementary file 1 [file life-15-00634-s001.zip › supp_table1.docx]

**Table S1**. Clinical criteria for COVID-19 severity classificaiton according to Croatian treatment guidelines used in this study [12].

| **Disease severity*** | **Criteria** |
| --- | --- |
| Mild | A patient presenting with symptoms of an uncomplicated respiratory tract infection, which may include fever, general malaise, headache, myalgia, rhinorrhea, sore throat, and/or cough.  There are no signs of dehydration, sepsis, or respiratory distress (dyspnea).  A child with an acute upper respiratory tract infection. |
| Moderate | An adult patient with more severe symptoms of illness and/or pneumonia, but without criteria for severe pneumonia, and without the need for supplemental oxygen therapy (SpO₂ >93% on room air).  A child with pneumonia, not meeting criteria for severe disease. |
| Severe | An adult patient with severe (bilateral) pneumonia accompanied by at least one of the following signs: respiratory rate >30 breaths/min, respiratory failure, or the need for supplemental oxygen therapy (SpO₂ ≤ 93% on room air).  A child with severe pneumonia, presenting with one of the following signs: central cyanosis or SpO₂ ≤ 90%, marked dyspnea, impaired general condition, altered consciousness, or seizures |
| Critical | An adult patient or child meeting criteria for ARDS**, sepsis, or septic shock, with or without acute dysfunction of other organ systems (shock, renal failure, coagulopathy, altered consciousness). |

SpO₂ = Peripheral capillary oxygen saturation, ARDS = Acute respiratory distress syndrome

* Immunocompromised patients should be classified one category higher (more severe disease)

** Definition of ARDS based on arterial blood gas (acid-base status): Mild ARDS: PaO₂/FiO₂ 200–300 mmHg, Moderate ARDS: PaO₂/FiO₂ 100–199 mmHg, Severe ARDS: PaO₂/FiO₂ ≤100 mmHg
