# Supplementary figures and images for: Prognostic Value of Biomarkers in COVID-19: Associations with Disease Severity, Viral Variants, and Comorbidities—A Retrospective Observational Single-Center Cohort Study
Source: Life (Basel). 2025 Apr 10;15(4):634. doi: 10.3390/life15040634 (PMC12028838; doi:10.3390/life15040634)

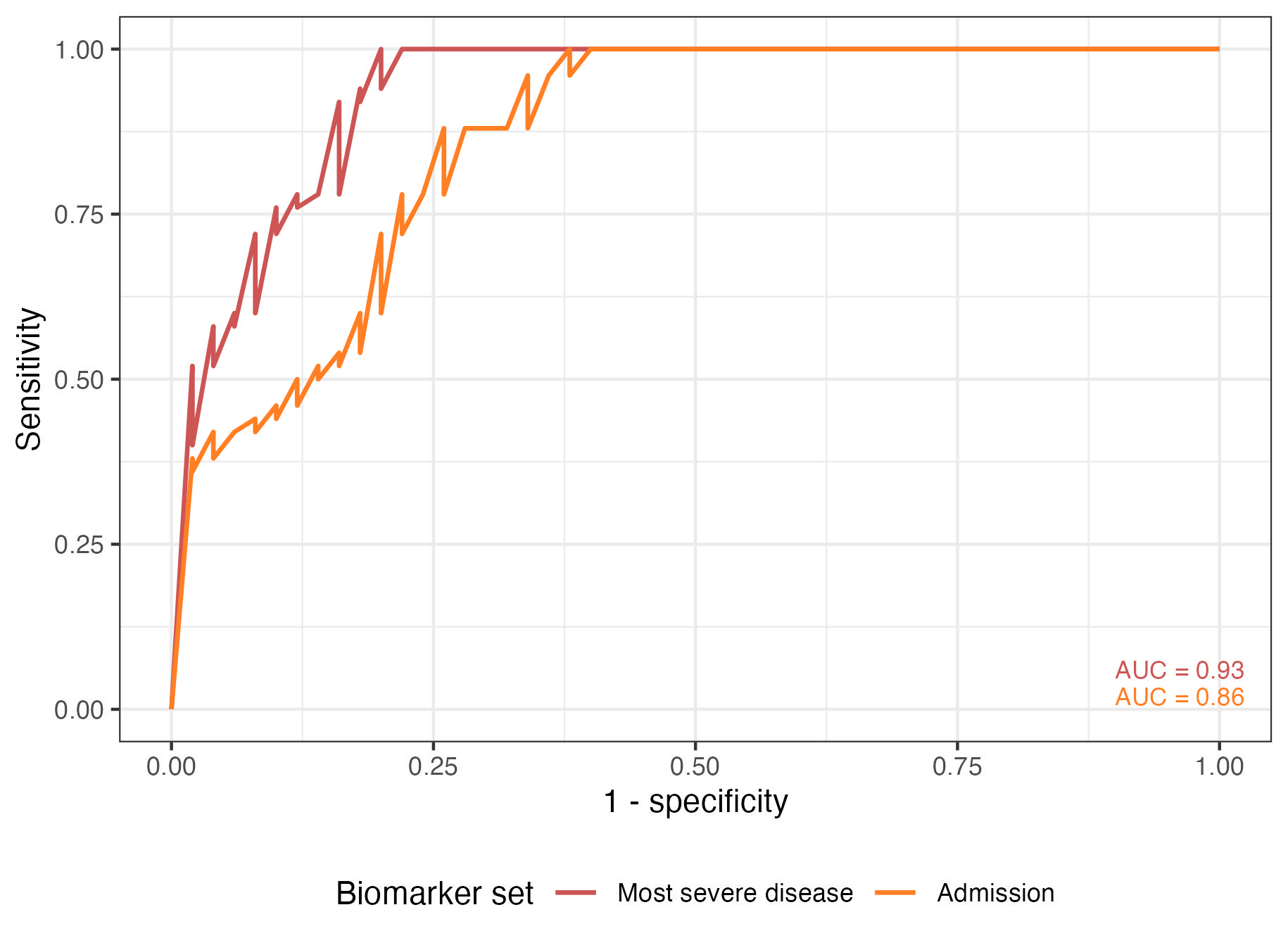

Supplement: Supplementary file 1 [file life-15-00634-s001.zip › sup_fig2.jpg]

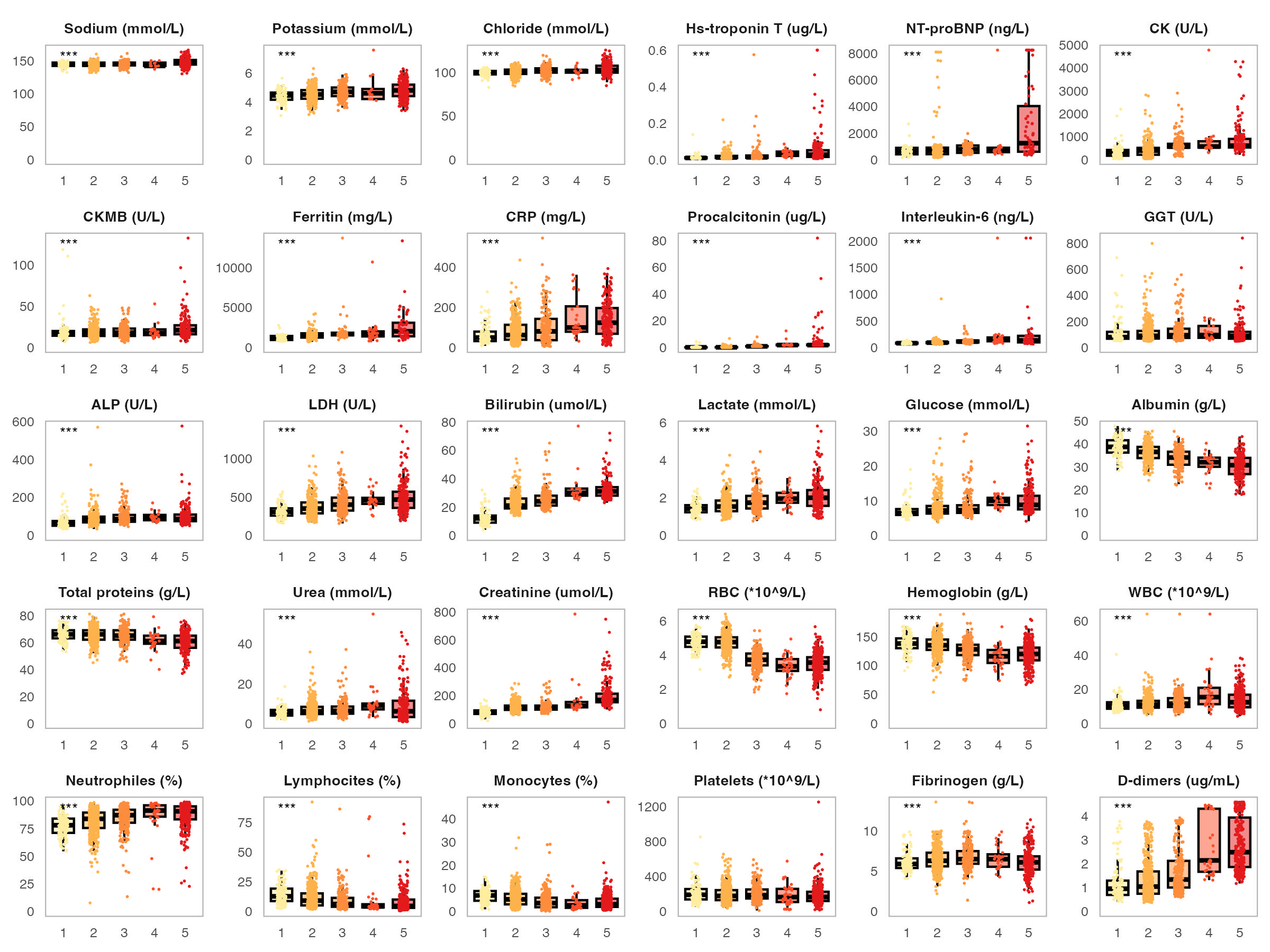

Supplement: Supplementary file 1 [file life-15-00634-s001.zip › sup_fig1.jpg]
